# Dengue virus infection in travellers after dengue vaccination, Germany 2023–24

**DOI:** 10.1093/jtm/taaf037

**Published:** 2025-05-03

**Authors:** Raskit Lachmann, Christina Frank, Hendrik Wilking, Kerstin Kling

**Affiliations:** Department for Infectious Disease Epidemiology, Robert Koch Institute, Seestrasse 10, 13351 Berlin, Germany; Department for Infectious Disease Epidemiology, Robert Koch Institute, Seestrasse 10, 13351 Berlin, Germany; Department for Infectious Disease Epidemiology, Robert Koch Institute, Seestrasse 10, 13351 Berlin, Germany; Department for Infectious Disease Epidemiology, Robert Koch Institute, Seestrasse 10, 13351 Berlin, Germany

## Abstract

After the introduction of the dengue vaccination in spring 2023, 48 cases of dengue infection with a dengue vaccination history were registered between September 2023 and December 2024 among 2079 German travellers with dengue infection returning from abroad.

Dengue virus (DENV) circulation has recently increased in many countries around the world with all four serotypes occurring in almost all endemic regions.^1^ In 2024, over 14 million dengue cases and over 10 000 dengue-related deaths have been reported worldwide and dengue was the most common arboviral illness that occurred in international travellers.^2^ Reported DENV infections in Germany occurred exclusively in travellers returning from dengue-endemic countries. In line with the global increase in dengue cases, the highest number of yearly cases ever, >1.600, was recorded among German travellers in 2024. Here, we report notified DENV infections in German travellers previously vaccinated against dengue with the Qdenga vaccine.

The vaccine Qdenga from Takeda was approved by the European Commission in December 2022 and has been available in Germany since March 2023.

Since November 2023, the German Standing Committee on Vaccination (STIKO) recommends a full vaccination series with two vaccine doses at a minimum interval of 3 months between the doses against dengue for German travellers. The general recommendation applies only for individuals who have a history of a laboratory-confirmed DENV infection and who are travelling to a dengue-endemic region with increased risk of exposure.^3^ This is in line with the WHO recommendation that persons, who have previously been infected with any of the 4 DENV serotypes, may benefit from the vaccination.^4^ In accordance with the EMA approval, individuals who have not yet experienced dengue can also be vaccinated. In individual instances of a dengue-naïve person being vaccinated, the STIKO recommends informing them that the risk of an increase in the severity of the disease cannot be ruled out in the event of a subsequent infection. The currently available data for dengue-naïve individuals could not demonstrate any protection against DENV-3 and -4-associated disease following vaccination. The risk of severe dengue is higher with a second infection than with a first infection, which is explained by an antibody-mediated increased severity of infection (antibody-dependent enhancement, ADE).^5^^,^^6^ This is the reason why the STIKO recommendation for travellers from Germany to endemic countries, in contrast to the EMA approval, makes a distinction between people who have already experienced dengue and people who are naive to dengue. It considers that the vaccination may prevent a second and potentially more severe DENV infection in people who have had an infection before the vaccination and that it cannot be ruled out that vaccinees who were dengue-naïve might face a higher ADE-risk when getting in contact with a wild virus later.

Diagnosed DENV infections in Germany are notifiable according to the Protection against Infection Act (IfSG). Dengue cases fulfilling the reference definition are individuals with laboratory-confirmed DENV infections (by single IgM detection, fourfold IgM/IgG increase, positive NS1-antigen test or positive PCR) and with at least fever as a symptom. Serological evidence is only accepted after a stay in a known dengue-endemic area before symptom onset. Positive laboratory tests for dengue clearly associated with a recent vaccination (e.g. titre tests) are not notifiable to avoid reports of vaccine-induced dengue-like clinical picture. Dengue cases who were vaccinated once or twice > 30 days before they developed dengue-compatible symptoms in connection with a trip to a dengue-endemic area were classified as possible DENV infection after vaccination.

We analysed all notified dengue cases in Germany between 1st September 2023 (when the first dengue cases with prior vaccination were notified) and 30th November 2024 (data as of 22 January 2025, Table 1). In these 15 months, 2079 cases were notified in Germany that fulfilled the reference definition. Of these, 48 cases (2.3%) reported having been vaccinated against dengue; 1451 cases (70%) reported not being vaccinated, and for 580 cases (28%) no information was available as to whether they had been vaccinated against dengue. For seven vaccinated cases, the duration between the vaccination and symptom onset was 30 days or less and for 10 cases, the date of vaccination or symptom onset was missing; these 17 cases were discarded from further analysis, since laboratory diagnostic tests for dengue might be positive due to the very recent dengue vaccination not representing an acute DENV infection.^7^^,^^8^

**Table 1:** Characteristics of notified dengue cases in Germany

Characteristic	Overall *N* = 2079^a^	1 dose *N* = 27^a^	2 doses *N* = 4^a^	Not vaccinated *N* = 1451^a^	Vaccination information missing *N* = 597^a^
**Sex**					
Female	1023 (49%)	10 (38%)	1 (25%)	731 (50%)	281 (47%)
Male	1053 (51%)	16 (62%)	3 (75%)	719 (50%)	315 (53%)
Unknown	3	1	0	1	1
**Age in years (Median IQR)**	36 (28, 52)	43 (31, 52)	41 (28, 53)	37 (28, 52)	34 (27, 52)
**Hospitalized**					
No	1371 (74%)	24 (96%)	3 (100%)	1025 (77%)	319 (67%)
Yes	471 (26%)	1 (4.0%)	0 (0%)	312 (23%)	158 (33%)
Unknown	237	2	1	114	120
**Severity**					
Uncomplicated dengue fever	2074 (~100%)	27 (100%)	4 (100%)	1448 (~100%)	595 (~100%)
Dengue hemorrhagic fever/dengue shock syndrome	4 (0.2%)	0 (0%)	0 (0%)	2 (0.1%)	2 (0.3%)
Death	1 (<0.1%)	0 (0%)	0 (0%)	1 (<0.1%)	0 (0%)
**Qdenga**	25 (1.2%)	18 (67%)	3 (75%)	0 (0%)	4 (0.7%)
**Laboratory methods**					
Fourfold IgM/IgG increase	216 (10%)	9 (33%)	2 (50%)	137 (9.4%)	68 (11%)
PCR, antigen test or viral culture	1255 (60%)	4 (15%)	1 (25%)	915 (63%)	335 (56%)
Single IgM detection	608 (29%)	14 (52%)	1 (25%)	399 (27%)	194 (32%)
**Exposure on continent**					
Africa	177 (8.5%)	4 (15%)	0 (0%)	129 (8.9%)	44 (7.4%)
Americas	746 (36%)	7 (27%)	2 (50%)	507 (35%)	230 (39%)
Asia	1144 (55%)	15 (58%)	2 (50%)	806 (56%)	321 (54%)
Australia	2 (<0.1%)	0 (0%)	0 (0%)	1 (<0.1%)	1 (0.2%)
Europe	3 (0.1%)	0 (0%)	0 (0%)	3 (0.2%)	0 (0%)
Unknown	7	1	0	5	1

^a^ *n* (%).

Among the 31 vaccinated cases analysed, 27 (87%) had received one vaccine dose pre-travel (18× Qdenga, 9× vaccine type unstated but very likely to be Qdenga as no other vaccine is available on the market in Germany). For them, the median lag between vaccination and symptom onset was 94 days (Min-Max: 59–113 days). The four cases (13%) who had received two vaccine doses (3× Qdenga, 1x unstated but very likely to be Qdenga) had lags of 40, 172 and 299 days (1 unknown, but at least 30 days), between the most recent dose and symptom onset. Elapsed time between the two doses was 92, 110, and 131 days (1 unknown).

Hospitalization was much lower among vaccinated cases: One case of the 27 with one vaccine dose was hospitalized due to a DENV infection (4%) compared to 23% hospitalization among the not vaccinated cases. There were no hospitalizations in the four cases with two doses of vaccine. It is important to note that hospitalization cannot always be equated with severe dengue. Depending on the individual patient, country context, hospital setting, etc., the reasons for hospitalization may vary.

Breakthrough infections, as we describe here, are not unexpected since the vaccine efficacy (VE) of two doses of Qdenga shows heterogenous efficacy depending on the DENV serotype: in licensing studies, the VE against laboratory-confirmed DENV infection was 62.2% (DENV-3), 72.2% (DENV-1) and 97.7% (DENV-2) in the first year after vaccination. The VE against DENV-4 could not be analysed due to the small number of DENV cases induced by DENV-4 in the licensing studies.^3^^,^^9^ For only one dose, the VE up to 3 months was 82.1% (95% CI: 66.2–90.5) in licensing studies.^10^ Our data shows/confirms that breakthrough infections after one and two vaccine doses are not uncommon.

Our analysis has several limitations. We do not know the absolute number of vaccinations given to travellers in Germany. Cases may also have been reported without the vaccination status (the information on vaccination is only self-reported by cases in Germany) leading to an underestimation of vaccinated dengue cases. Vaccinated travellers may be more likely to seek health care because they have a higher general health awareness or are less likely to seek care as they assume that they are protected from DENV due to the vaccination. Laboratory diagnostics of notified cases in Germany often still rest on a single increased IgM titre; due to the passive collection of the surveillance data it was not possible to confirm the potential breakthrough infections by further laboratory diagnostics. And there was no information available about prior dengue infections or the severity of disease. Therefore, it was not possible to analyse whether infections after vaccination were more or less severe than infections in unvaccinated people.

Nonetheless, our data is comparable to the geo-sentinel data collected between 2007 and 2022 (before the vaccine became available) from international travellers returning from endemic setting (*n* = 5958), where they reported a similar proportion of hospitalized cases (26.7%) and proportion with severe dengue (0.5%).^1^

Given the availability of Qdenga on the German market, the recent increase in the number of dengue cases worldwide and the German population’s great willingness to travel, we are probably among the first dengue-non-endemic countries to notice DENV infections in vaccinated travellers. In the USA, the manufacturing company has withdrawn its application for approval from the FDA until more post-marketing effectiveness data is available, so no comparable data can be expected from American travellers in the near future. Our data underscores the importance of notifying travellers and medical professionals about the potential of symptomatic DENV infections occurring after vaccination, also after only one dose, to ensure informed decision-making. This is especially important as the vast majority of travellers are seronegative. In general, it should be ensured that the indication for vaccination is made correctly according to the STIKO recommendation, and complement the information that two doses are required for optimal protection. In those who are travelling with only one dose, the series should be completed after the travel with a minimum interval of 3 months between vaccine doses. Additionally, travellers should be reminded that even after vaccination good mosquito protection is essential to protect against DENV and other arthropod-borne infections.
